# Comparative Evaluation of Orthodontically-Induced Root Resorption Using Cone Beam Computed Tomography (CBCT) and Orthopantomogram (OPG) During En-Masse Retraction of Maxillary Anterior Teeth

**DOI:** 10.7759/cureus.31219

**Published:** 2022-11-07

**Authors:** Ahmed Alassiry

**Affiliations:** 1 Preventive Dental Sciences, Faculty of Dentistry, Najran University, Najran, SAU

**Keywords:** saudi arabia, bimaxillary protrusion, orthodontics, opg, cbct, apical root resorption

## Abstract

Background/Purpose: Orthodontically induced apical root resorption is an inescapable consequence of fixed orthodontics. This root resorption causes post-orthodontic complications in some treated cases. To avoid these complications proper diagnosis of the site and amount of resorption is important. The aim of this study was to compare the diagnostic ability of Orthopantomogram (OPG) and Cone Beam Computed Tomography (CBCT) in detecting apical root resorption after en-masse retraction of maxillary anterior teeth in a sample of Saudi Arabian population.

Materials and methods: The study sample comprised of 30 patients diagnosed with bimaxillary dentoalveolar protrusion. The treatment plan involved extraction of all first premolars followed by en-masse retraction of the anterior teeth. OPG images were obtained at the beginning of treatment and after the end of the retraction phase and a CBCT image were also obtained for the same patient at the same centre other than orthodontic reason like implant placement, temporomandibular joint (TMJ) problems, sleep apnea, etc. after orthodontic treatment completion. The scoring system of Levander and Malmgren was used to assess the degree and severity of root resorption in the maxillary incisors. Dalhbergs error and coefficient of reliability (ICC) were used to calculate the correlation between the two sets of readings. Pearson chi-square test was used to compare the difference in root resorption between OPG and CBCT images. A *P*-value of <0.05 was considered to be statistically significant.

Results: No resorption was observed in 39.2% and 16.6% of incisors with OPG and CBCT respectively. Mild resorption was observed in 50% and 66.7% of incisors with OPG and CBCT respectively. Moderate resorption was found in 10% and 15% of incisors with OPG and CBCT respectively. Severe root resorption was found in 0.8% and 1.7% of incisors with OPG and CBCT respectively. Statistically, significant differences were found in both methods of evaluation in all grades of root resorption for all the maxillary incisors (*P*<0.05).

Conclusion: OPG had consistently underestimated the amount of orthodontically induced apical root resorption when compared to CBCT. OPG is only useful for the primary evaluation of root resorption. CBCT can be used as an adjunct diagnostic tool on a case-to-case basis in patients with moderate to severe root resorption to manage post-orthodontic treatment complications.

## Introduction

External apical root resorption (EARR) is an undesirable yet common, indisputable, and uncontended phenomenon in modern orthodontic practice [1]. EARR histologically represents the demineralization of cementum and dentine around the apical root region and anatomically reflects a permanent loss of tooth structure around the root apex resulting in the physical shortening of root length [2,3]. Histologic studies have shown that more than 90% of root resorption occurs due to orthodontic forces whereas in radiological studies the incidence of EARR ranges from 5-18% [4,5].

Multiple and complex factors are responsible for the occurrence of apical root resorption. Risk factors can be internal or patient-related and external or treatment-related. Internal factors include systemic diseases, genetic predisposition, gender, age, alveolar bone density, root morphology, type of malocclusion, root proximity to cortical plate, previous endodontic treatment, and parafunctional habits [6]. External factors are directly related to the orthodontic treatment such as duration of treatment, type of appliance and mechanotherapy, the magnitude, direction, and type of forces, the distance of tooth movement, extraction of premolars, and root torqueing [7,8].

Definitive and decisive diagnoses of EARR can be obtained by periapical radiographs, orthopantomographs (OPG), subtraction radiographs, light microscopes, scanning electron microscopes, and cone-beam computed tomography (CBCT) [9]. These radiographic techniques tend to either underestimate or overestimate the amount of root resorption [10]. There is no gold standard diagnostic method to detect orthodontically induced apical root resorption. Two-dimensional methods such as periapical and panoramic radiographs or OPG are associated with superimposition, image distortion, magnification error, and reliability issues [11]. Moreover, these methods cannot quantify the volume of root loss and are also unable to differentiate between buccal and lingual root resorption [12]. Whereas three-dimensional methods such as cone-beam computed tomography overcome these shortcomings and enable superior qualitative assessment [13]. Nevertheless, these are associated with higher radiation dosage, training, and machine cost [14].

Bimaxillary dentoalveolar protrusion is a common malocclusion with a prevalence of around 3-68% in the general population [15]. Treatment usually involved extraction of first premolars followed by retraction of anterior teeth into the extraction spaces. Studies have shown that the duration of active orthodontic forces and the distance of the teeth moved are the most important potential risk factors for the occurrence of EARR [16,17]. En-masse retraction of anterior teeth is time-consuming and necessitates significant movement of the incisors through the alveolar bone. Thus, root resorption is imminent and inevitable during the complete course of the treatment. Hence, it is clinically important to be familiar with efficient and effective methods to diagnose root resorption. The aim of this article was to compare the diagnostic ability of OPG and CBCT in detecting orthodontically induced apical root resorption after en-masse retraction of maxillary anterior teeth in a sample of the Saudi Arabian population.

## Materials and methods

The present retrospective study was conducted after approval by the Scientific Research Ethical Committee (IRB Reference No.:444-40-13520-DS), Najran University, Najran, Saudi Arabia. Data from 30 patients who had completed the orthodontic treatment at the Sama Dental Centre, Najran, Saudi Arabia, were included in the study by considering the following inclusion and exclusion criteria. Inclusion criteria: 1. Patient of 15-25 years of age of Saudi Arabian origin, 2. Patients with no significant medical history, 3. Patients with bimaxillary dentoalveolar protrusion with less than 3 mm of crowding, 4. Patients with the duration of en-masse retraction between 6-8 months, 5. Complete orthodontic treatment was done by the same orthodontist., 6. The patient had both pre-operative and post-operative good quality OPG, 7. Patient having a CBCT after completion of orthodontic treatment for implant placement. Exclusion criteria: 1. Patients with systemic diseases, bony disorders, and chronic use of anti-inflammatory drugs, 2. Patients with a previous history of orthodontic treatment, dental trauma, or EARR, 3. Patients with parafunctional habits like bruxism and endodontically treated teeth.

Orthodontic treatment protocol for all the patients was bonding with metal MBT (MacLaughlin, Bennet, and Trevisi) prescription of 0.022 x 0.028 inch (Ortho Organizers, Carlsbad, California, USA) and Transbond XT (3M Unitek, Monrovia, California, USA). All the indicated first premolars were extracted and alignment and levelling were performed using 0.014-0.016 inch NiTi arch-wires (TrueFlex, Florida, USA). This progressed to 0.019 x 0.025-inch stainless steel working wires with fit passively into the bracket slot. A transpalatal arch (TPA) appliance was bonded on all the patients to reinforce the anchorage. After initial alignment and levelling, en-masse retraction of maxillary anterior teeth was performed using sliding mechanics. The six maxillary anterior teeth were consolidated by the figure of 8 ligatures. NiTi close coil springs (G&H Wire, Franklin, Indiana, USA) of either 9 or 12 mm length were used from canine to molar to retract the maxillary anteriors with a constant force of 150 g on both sides. The CBCT image was acquired using a CBCT instrument (Veraview FX800, Morita, California, USA). The exposure parameters for CBCT scans were 66 kVp, 10 mA, scanning time 16 seconds, and voxel size 300.

The scoring system of Levander and Malmgren was used to assess the degree and severity of root resorption in the panoramic and CBCT images [18]. The following categorization of root resorption in five grades was used: Grade 0: No root resorption, Grade 1: Mild resorption, with the root of normal length and only an irregular contour, Grade 2: Moderate resorption, with small areas of root loss and the apex having an almost straight contour, Grade 3: Severe resorption, with loss of almost one-third of root length, Grade 4: Extreme resorption, with loss of more than one-third of the root length.

A single-experienced examiner assessed the panoramic and CBCT images separately from each patient and categorized them into their respective degree of root resorption using the scoring system. The images were re-examined after a four-week interval by the same investigator to assess the intra-examiner reliability. The data were analyzed using SPSS for Windows, version 20.0 (IBM Corp., Armonk, NY). Dalhbergs error and coefficient of reliability (ICC) were used to calculate the correlation between the two sets of readings. Pearson Chi-square test was used to statistically evaluate the difference in root resorption between OPG and CBCT images. A P-value of <0.05 was considered to be statistically significant. 

## Results

A total of 120 maxillary incisor teeth from 30 patients were evaluated in the study. The sample comprised 12 male and 18 female patients with mean±SD age of 17.55±4.68 years. The inter-class correlation coefficient (ICC) found a high correlation (>0.9) between two sets of scoring for OPG and CBCT images. No statistical difference was found in the two sets of readings (P>0.05).

Grading of apical root resorption in maxillary right central incisor (11), maxillary right lateral incisor (12), maxillary left central incisor (21), and maxillary right lateral incisor (22) as seen in OPG and CBCT images after completion of retraction phase is shown in Table 1. Grading of all the teeth with different scores of root resorption as assessed by OPG and CBCT images is shown in Table 2. Statistically, a significant difference was found in both methods of evaluation in all the grades of root resorption for all the maxillary incisors (P<0.05) (Tables 1 and 2). OPG had consistently underestimated the amount of root resorption in all the maxillary incisors when compared to CBCT. Around 39.2% of incisors had no resorption as observed in OPG whereas 16.6% had no resorption when evaluated with CBCT images. Mild resorption was observed in 50% of incisors with OPG and in 66.7% with CBCT. Moderate root resorption was found to be 10% with OPG and 15% with CBCT. Severe root resorption was found at 0.8% with OPG and 1.7% with CBCT. Extreme root resorption was not found in any of the evaluated incisors with both imaging techniques.

**Table 1:** Grading of root resorption in maxillary anterior teeth after completion of retraction by OPG and CBCT OPG: Orthopantomogram; CBCT: Cone Beam Computed Tomography

Maxillary Tooth		Grading of root resorption (N/ %)					P-Value
		0	1	2	3	4	
11	OPG	16 (53.3%)	11 (36.6%)	2 (6.6%)	1 (3.3%)	0 (0%)	0.0215*
	CBCT	10 (33.3%)	17 (56.6%)	2 (6.6%)	1 (3.3%)	0 (0%)	
21	OPG	17 (56.6%)	12 (40%)	1 (3.3%)	0 (0%)	0 (0%)	0.0463*
	CBCT	10 (33.3%)	16 (53.3%)	3 (10%)	1 (3.3%)	0 (0%)	
12	OPG	11 (36.6%)	14 (46.6%)	4 (13.3%)	1 (3.3%)	0 (0%)	0.0343*
	CBCT	5 (16.6%)	17 (56.6%)	6 (20%)	2 (6.6%)	0 (0%)	
22	OPG	10 (33.3%)	13 (43.3%)	5 (16.6%)	2 (6.6%)	0 (0%)	0.0196*
	CBCT	4 (13.3%)	17 (56.6%)	6 (20%)	3 (10%)	0 (0%)	

**Table 2:** Grading of root resorption in all the maxillary incisor teeth by OPG and CBCT OPG: Orthopantomogram; CBCT: Cone Beam Computed Tomography

CBCT (N/%)	OPG (N/%)					Total (N/%)
	0	1	2	3	4	
0	4 (3.3%)	15 (12.5%)	1 (0.8%)	0 (0%)	0 (0%)	20 (16.6%)
1	33 (27.5%)	40 (33.3%)	6 (5%)	1 (0.8%)	0 (0%)	80 (66.7%)
2	9 (7.5%)	4 (3.3%)	5 (4.1%)	0 (0%)	0 (0%)	18 (15%)
3	1 (0.8%)	1 (0.8%)	0 (0%)	0 (0%)	0 (0%)	2 (1.7%)
4	0 (0%)	0 (0%)	0 (0%)	0 (0%)	0 (0%)	0 (0%)
Total (N/%)	47 (39.2%)	60 (50%)	12 (10%)	1 (0.8%)	0 (0%)	120 (100%)

## Discussion

The scope of orthodontic treatment is expanding as the techniques are improving day by day. Likewise, patients’ expectations and understanding regarding the treatment are also increasing. Hence, it is well judged for an orthodontist to be aware of orthodontically induced apical root resorption. Currently, there are several methods to diagnose apical root resorption in a clinical setup. Nevertheless, there is no standard benchmark diagnostic method for the same. Most of the diagnosis revolves around the use of OPG and CBCT methods owing to their availability in an institutional, educational or diagnostic establishment. In this study, we have compared the diagnostic ability of OPG and CBCT in detecting orthodontically induced apical root resorption after en-masse retraction of maxillary anterior teeth in the Saudi Arabian population. Around 61% of teeth showed root resorption with OPG whereas 83% of teeth were diagnosed with root resorption when evaluated with CBCT. For all the scoring and grading for root resorption, OPG images had consistently underestimated the amount of root resorption when compared to CBCT.

The findings of this study are in agreement with the results published by various investigations to evaluate the efficiency of OPG and CBCT for apical root resorption. According to Dudic et al., 44% of teeth showed root resorption with OPG and 69% with CBCT [19]. On the contrary, Deng et al. found that two-dimensional radiographic data may overestimate the amount of root resorption [20]. According to Chan and Darendeliler, two-dimensional techniques are useful but should not be used for the quantitative evaluation of root resorption [21]. Maret et al. supported the use of CBCT for the volumetric measurement of root resorption [22]. Despite the fact that some clinically acceptable amount of root resorption occurs in every orthodontic patient, CBCT is not recommended for each one of them. American Dental Association recommends CBCT only where there is expected diagnostic benefit or significant improvement in the clinical outcome [23]. Heimisdottir et al. advocated the use of CBCT scans to accurately judge the severity of root resorption only in moderately to severely absorbed roots [24].

Different degrees of root resorption invariably occur in all types of orthodontic treatment, fortunately, they are clinically acceptable. Severe root resorption involving more than 4 mm or one-third of root length involvement has been observed in only 1-5% of the teeth [18]. According to Remington et al., most of the root resorption is seen in maxillary incisors as they are the farthest-moved teeth clinically [25]. In a study conducted by Mcnab et al., 3.72 times more root resorption was found in extraction cases in compared to non-extraction patients [26]. Root resorption usually terminates to progress with the end of active orthodontic treatment. Radiographic records have shown that over time reparative healing of the root tips and smoothing of the ends occurs. Conflicting reports have been observed regarding the fate of severely resorbed teeth after orthodontic treatment [27]. Severe resorption may threaten the vitality of the tooth, cause mobility or provide low resistance to masticatory functional loads [28]. When in fact long-term observational studies have shown that severely resorbed teeth after orthodontic treatment continues to function in a reasonable manner with no tooth loss or mobility [29].

Histologic studies have found a 100% frequency of root resorption in orthodontically treated teeth [30]. Since these studies are performed on extracted teeth, they cannot be used routinely for practical purposes. Conventional two-dimensional radiographic methods like periapical x-rays and orthopantomograms are easily available to an orthodontist for any preliminary investigations. OPG is relatively inexpensive and involves less radiation exposure but is associated with low-quality images, magnification, and distortion of images [31]. On the contrary, CBCT machines are expensive and radiation is usually more than most than effective doses of panoramic imaging [14]. However, they provide the best images with minimum radiation dosage. Since their usage means additional financial burden and risk of radiation exposure to the patient, their recommendations should be made on a case-to-case basis considering the cost-benefit ratio. CBCT for evaluating root resorption can be employed for research purposes and assessing high-risk patients with greater predisposition during orthodontic treatment [19]. CBCT has been found to be a valuable tool in diagnosing root resorption associated with impacted maxillary canines [32,33]. In such indicated cases, periodic assessment and monitoring may limit the progression of orthodontic treatment-induced root resorption.

The limitation of the study lies in the fact that root resorption and repair are continuous processes whereas root resorption was observed at one point in time. Long-term studies are recommended to understand the resorption-repair-regeneration process. Also, the sample size was less in the study due to concerns about excessive radiation exposure to the patients. Further studies with increased sample size and incorporation of other patient-related factors affecting root resorption are recommended in the future.

## Conclusions

Root resorption may be inevitable but its accurate diagnosis is not. This study has shown that the OPG tends to underestimate the amount of root resorption whereas CBCT provides more explicit, precise, and valuable information regarding the quality and quantity of apical root resorption. However, the authors recommend judicious use of CBCT in apical root resorption cases where the diagnosis will bring significant clinical value in determining the outcome and future course of treatment. The authors recommend the use of OPG for the primary evaluation of root resorption. In cases with moderate to severe root resorption, findings should be correlated clinically and CBCT scans should be performed accordingly to evaluate the resorption three-dimensionally. The present study is based on sample size and hence, further research is needed with large sample sizes.
